# Supplementary material for: Distinct dynein complexes defined by DYNLRB1 and DYNLRB2 regulate mitotic and male meiotic spindle bipolarity
Source: Nat Commun. 2023 Mar 27;14:1715. doi: 10.1038/s41467-023-37370-7 (PMC10042829; doi:10.1038/s41467-023-37370-7)
Supplement: Supplementary file 1 — Supplementary Information [file 41467_2023_37370_MOESM1_ESM.pdf]

Supplementary Information

Distinct dynein complexes defined by DYNLRB1 and DYNLRB2 regulate mitotic and male meiotic spindle bipolarity

He *et al.*

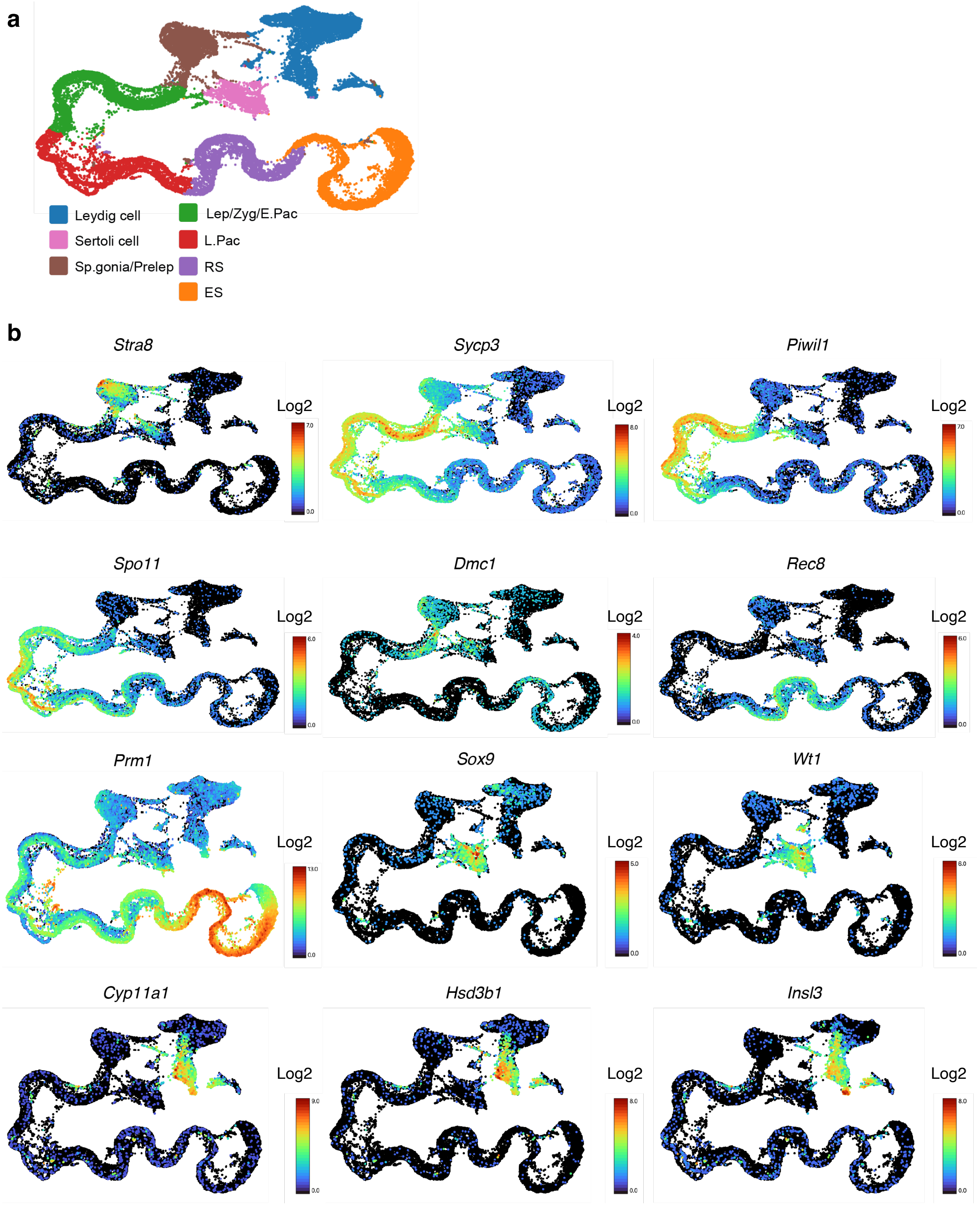

Supplementary Fig. 1 Single-cell RNA sequencing analysis of mouse testis.
(**a**) Colors represent the cell types of clustered cell populations.
(**b**) UMAP plots showing the expression patterns of known marker genes for spermatogenic stages with color gradients for their expression level in log2 arbitrary units. *Stra8*: spermatogonia and preleptotene spermatocytes, *Sycp3*, *Piwil1*, *Spo11*, *Dmc1*: meiotic spermatocytes, *Rec8*: dividing spermatocytes and round spermatocytes, *Sox9* and *Wt1*: Sertoli cells, *Cyp11a1*, *Hsd3b1*, *Insl3*: Leydig cells. Spermatogonia (Sp.gonia), preleptotene (Prelep), leptotene (Lep), zygotene (Zyg), early-pachytene (E.Pac), late-pachytene (L.Pac), round spermatid (RS), elongated spermatid (ES).

a

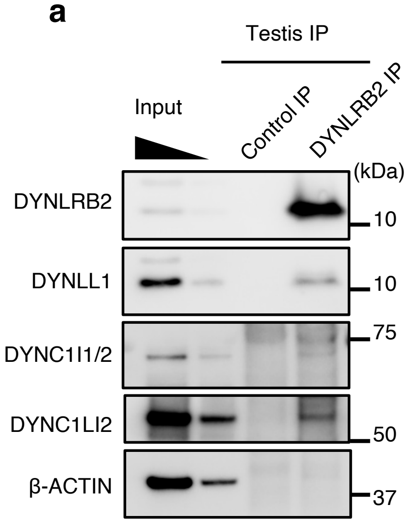

b

Gene	# of clone	Gene function
Wdr34	31	Dynein-2 intermediate chain
Dync1i1	29	Dynein-1 intermediate chain
Dync1i2	26	Dynein-1 intermediate chain
Ccdc45 (Cep95)	5	Centriolar satellite
Prc1	2	Cytokinesis, central spindle and midbody
Cenpe	1	Kinetochore and spindle
Pcm1	1	Centriolar satellite

c

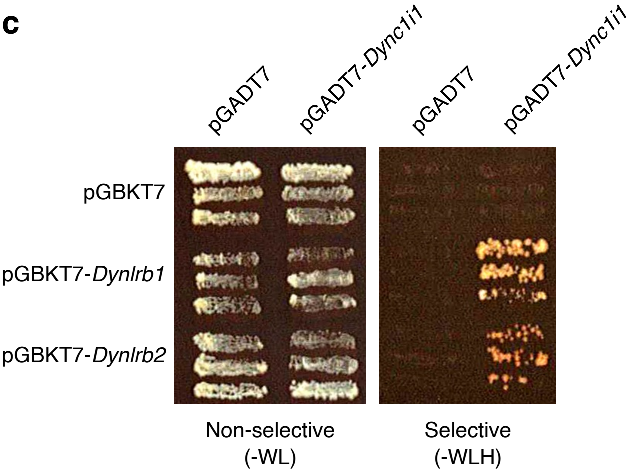

Supplementary Fig. 2 DYNLRB2 forms functional dynein complexes.
(**a**) IPs from mouse testis extracts with the indicated antibodies.
(**b**) Genes identified in the DYNLRB2 yeast two-hybrid screening. Only genes that have roles in mitotic spindle regulation are shown.
(**c**) Yeast two-hybrid interactions. DYNC1I1 was used as prey, and DYNLRB1 and DYNLRB2 were used as bait.

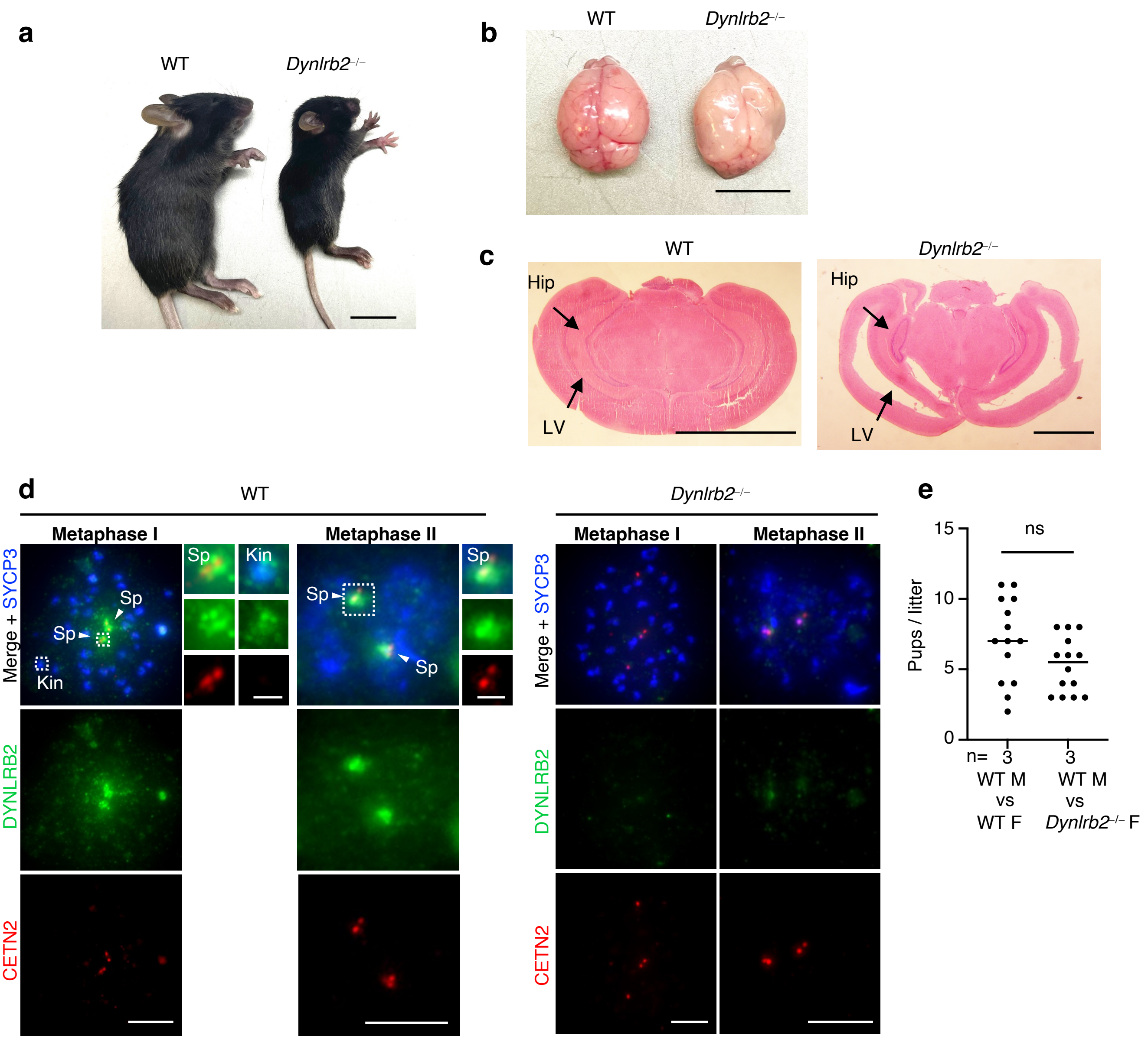

Supplementary Fig. 3 Somatic defects in *Dynlrb2*$^{-/-}$ juvenile mice.
(**a**) WT and *Dynlrb2*$^{-/-}$ littermates at PD18. Some juvenile *Dynlrb2*$^{-/-}$ mice had smaller body size compared to the WT littermates and developed hydrocephalus. Scale bar: 1 cm.
(**b**) Brain samples from WT and *Dynlrb2*$^{-/-}$ littermates at PD18. Scale bar: 1 cm.
(**c**) Brain section from WT and *Dynlrb2*$^{-/-}$ littermates at PD18 stained with hematoxylin and eosin. Hippocampus (Hip), lateral ventricle (LV). Scale bar: 250 µm.
(**d**) Immunostaining of WT and *Dynlrb2*$^{-/-}$ spermatocytes. Spindle pole (Sp), kinetochore (Kin). The spindle pole signals and kinetochore signals with intensified DYNLRB2 are shown in magnified panels. Both spindle pole and kinetochore signals completely disappeared in the *Dynlrb2*$^{-/-}$ spermatocytes. Scale bar: 5 µm (1 µm, magnified panel).
(**e**) The average number of pups per litter. PD60 male (M) and female (F) mice with indicated genotypes were paired for more than 90 days of continuous breeding. n indicates the number of mating pairs examined. All analyses used two-tailed *t*-tests. ns: not significant.

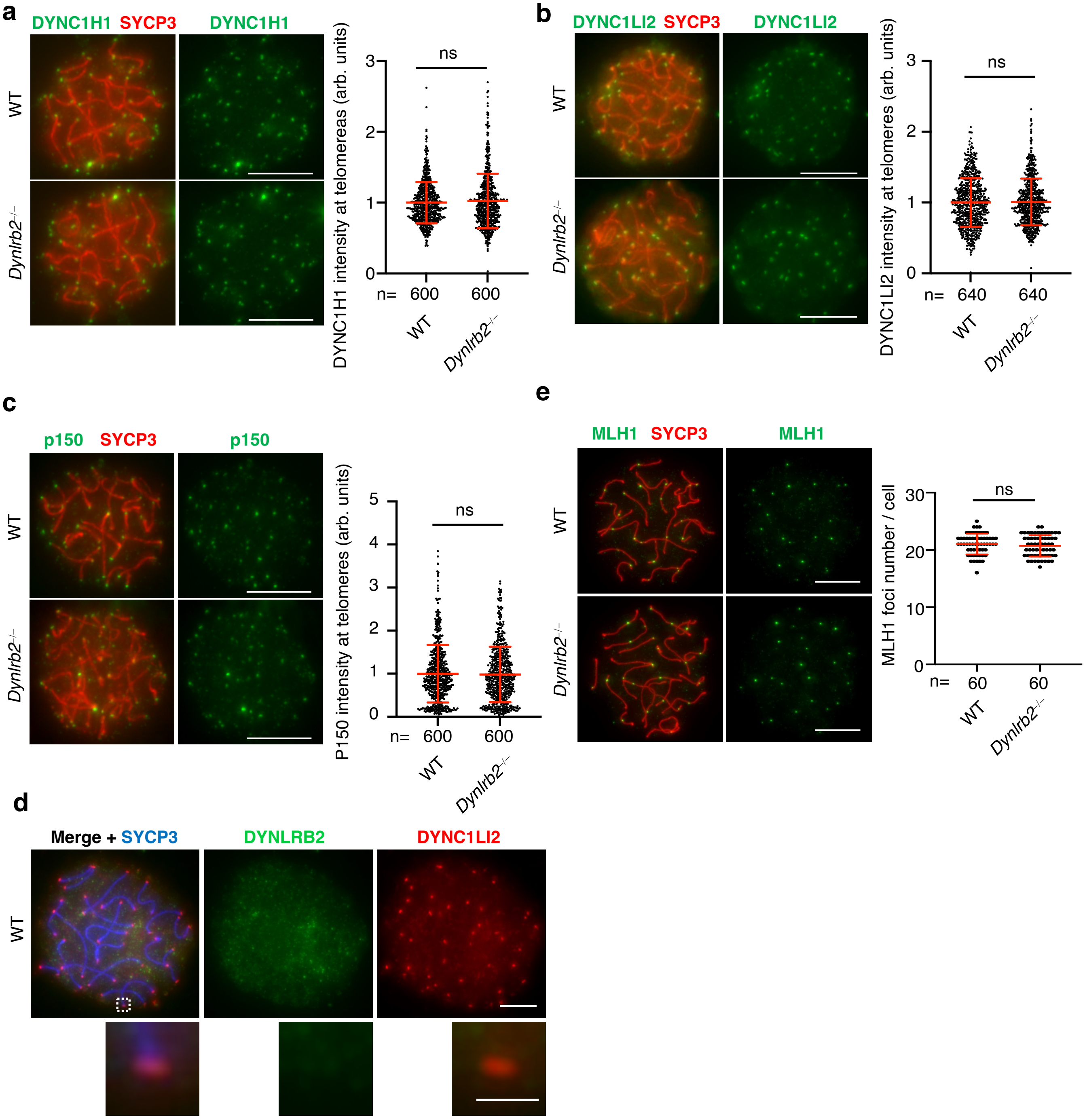

Supplementary Fig. 4 DYNLRB2 is dispensable for dynein function at meiotic telomeres in prophase I.
(**a-c**) Immunostaining of pachytene spermatocytes stained with the indicated antibodies. The graphs show the quantification of DYNC1H1 (A), DYNC1LI2 (B), and p150 (C) signal intensity at telomeres normalized to the average value of the controls. The mean values with SD are shown, and n shows the total number of telomeres collected from more than 30 pachytene cells pooled from three independent experiments using three different mice. Scale bar: 5 µm.
(**d**) Immunostaining of WT pachytene spermatocytes. A telomeric signal is shown in the magnified panel. DYNC1LI2 but not DYNLRB2 formed foci at telomeres. Scale bar: 5 µm (1 µm, magnified panel).
(**e**) Immunostaining of late-pachytene spermatocytes with SYCP3 in red and MLH1, a destined crossover marker, in green. The graphs show the quantification of MLH1 foci. The mean values with SD are shown, and n shows the total number late-pachytene spermatocytes pooled from three independent experiments using three different mice. There was no significant difference in the MLH1 foci number between WT and *Dynlrb2*$^{-/-}$, suggesting that the progression of meiotic recombination was normal in *Dynlrb2*$^{-/-}$ spermatocytes. Scale bar: 5 µm (1 µm, magnified panel). All analyses used two-tailed *t*-tests. ns: not significant.

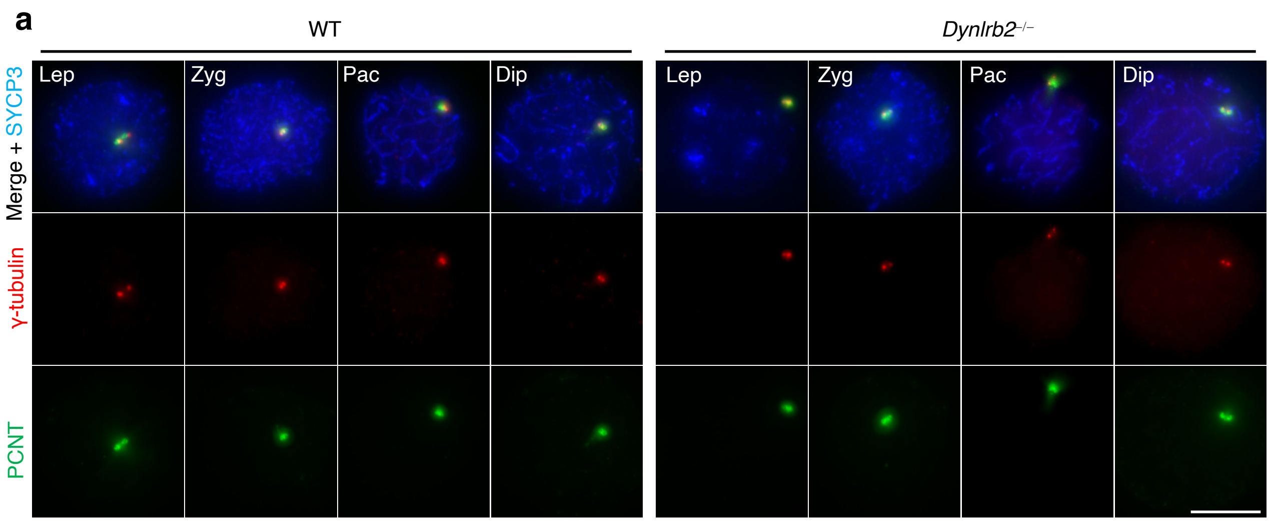

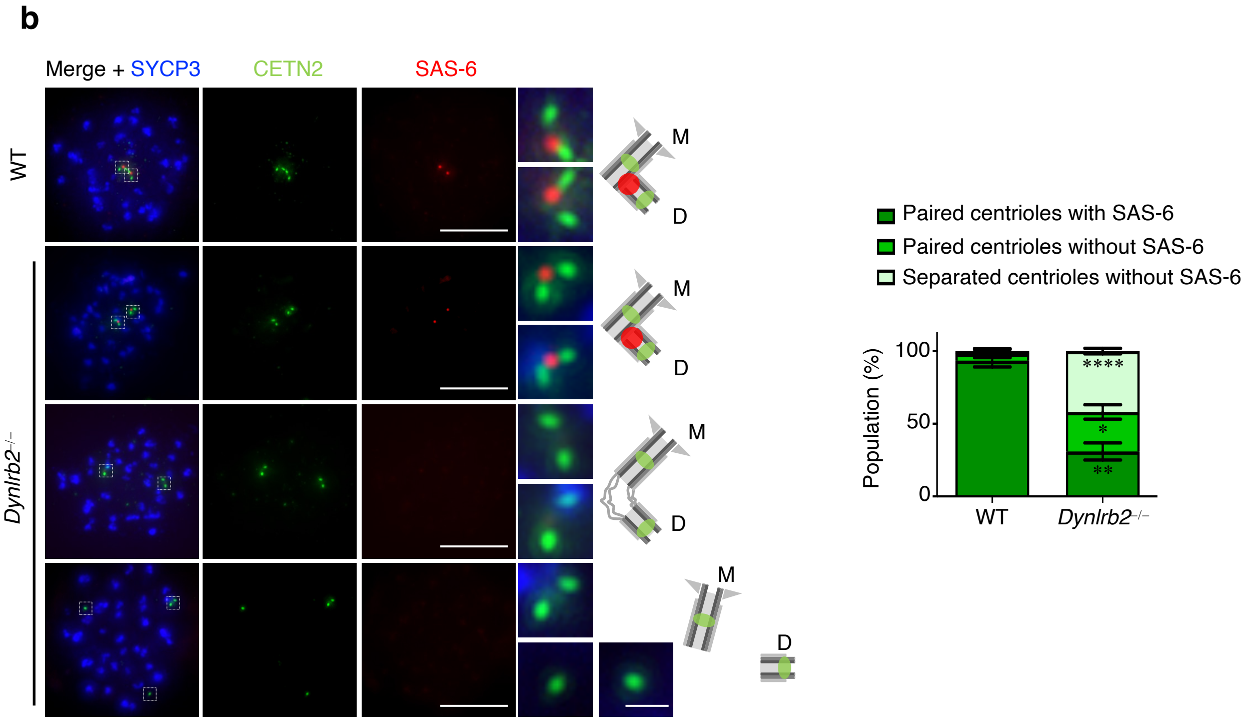

Supplementary Fig. 5 Premature centriole disengagement in *Dynlrb2*$^{-/-}$ metaphase I spermatocytes.
(**a**) Spermatocyte chromosome spreads from WT and *Dynlrb2*$^{-/-}$ males stained with the indicated antibodies and DAPI. Lep, leptotene; Zyg, zygotene; Pac, pachytene; Dip, diplotene. A tightly paired PCNT and γ-tubulin signal was consistently observed throughout prophase I in both WT and *Dynlrb2*$^{-/-}$ spermatocytes during prophase I, suggesting that PCM fragmentation does not occur at this stage. Scale bar: 5 µm.
(**b**) Immunostaining of metaphase I spermatocytes. The graph shows the quantification of centriolar defects. The mean values with SD of three independent experiments using three different mice are shown (n = 61 and 185 cells for WT and *Dynlrb2*$^{-/-}$, respectively). Scale bar: 5 µm (0.5 µm, magnified panel). All analyses used two-tailed *t*-tests. *$p < 0.05$,**$p < 0.01$, ****$p < 0.0001$.

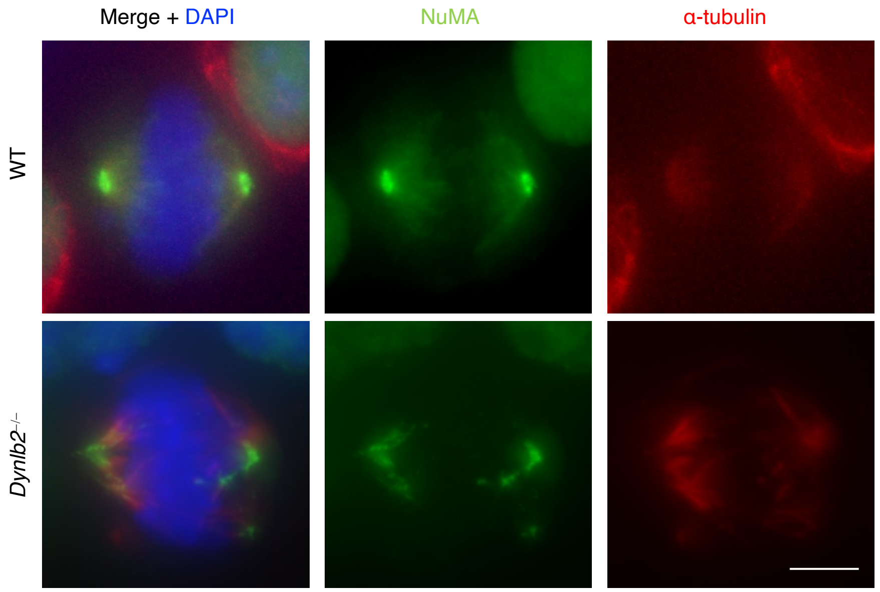

Supplementary Fig. 6 NuMA failed to accumulate at the spindle poles in *Dynlrb2*$^{-/-}$ metaphase I spermatocytes. Immunostaining of metaphase I *Dynlrb2*$^{-/-}$ spermatocytes prepared by the squash technique showing the reduction of total signal intensity and the lack of accumulation of NuMA at the spindle poles. Scale bar: 5 µm.

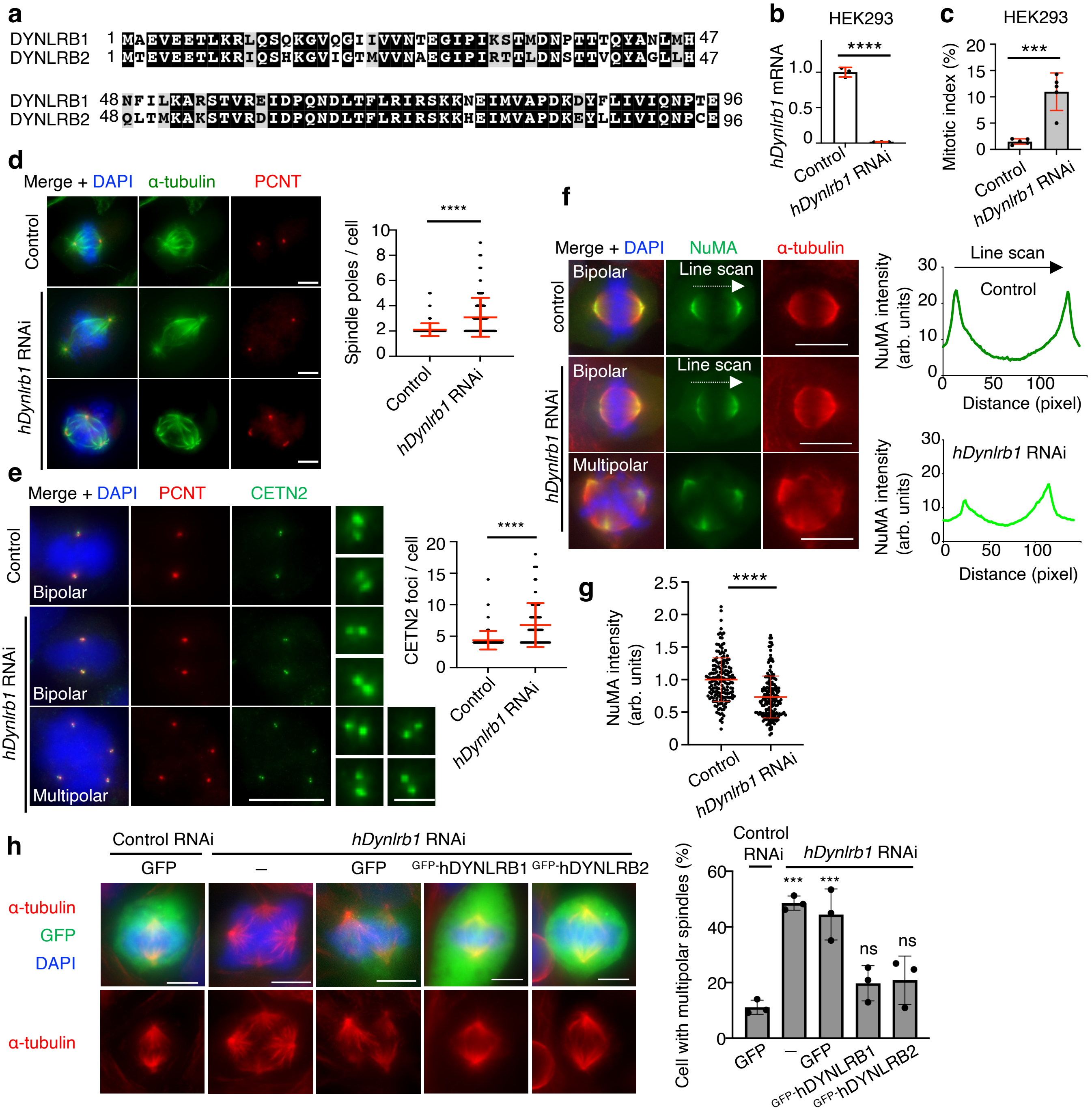

Supplementary Fig. 7 hDYNLRB1 is indispensable for spindle bipolarity in HEK293 cells.

(**a**) Sequence alignment of mouse DYNLRB1 and DYNLRB2. Sequence data are from the NCBI protein database.

(**b**) The quantification of h*DYNLRB1* mRNA expression level normalized to the average value of controls in HEK293 cells. The mean values with SD of three independent experiments are shown.

(**c**) The quantification of the mitotic cell population in HEK293 cells. The mean values with SD of five independent experiments are shown (n = 1,742 and 911 cells for control and *Dynlrb1* RNAi, respectively).

(**d**) Immunostaining of HEK293 cells in metaphase. The graph shows the quantification of spindle pole number. Mean values with SD are shown. Data were pooled from three independent experiments for each condition (n = 90 metaphase cells for both conditions). Scale bar: 5 µm.

(**e**) Immunostaining of HEK293 cells at metaphase. The graph shows the quantification of CETN2 foci number. Mean values with SD are shown. Data were pooled from three independent experiments for both conditions (n = 72 metaphase cells for both conditions). Scale bar: 5 µm (0.5 µm, magnified panel).

(**f**) Immunostaining of HEK293 cells at metaphase. The graphs show the line scan analysis of NuMA intensity between each spindle pole. Scale bar: 5 µm.

(**g**) The quantification of NuMA signal intensity at metaphase in HEK293 cells normalized to the average value of the controls. The mean values with SD are shown. Data were pooled from three independent experiments (n = 176 and 182 spindle poles for control and h*DYNLRB1* RNAi, respectively). Only cells that formed bipolar spindles were quantified.

(**h**) Immunostaining of HEK293 cells at metaphase. The graph shows the frequency of metaphase cells that formed multipolar spindles. The mean values with SD of three independent experiments are shown (more than 300 metaphase cells were quantified for each condition). Scale bar: 5 µm.

Two-tailed *t*-tests were used in (**b**), (**c**), (**d**), (**e**), and (**g**). One-way ANOVA with Dunnett's multiple comparisons test was used in (**h**). ns: not significant, ***$p < 0.001$, ****$p < 0.0001$.

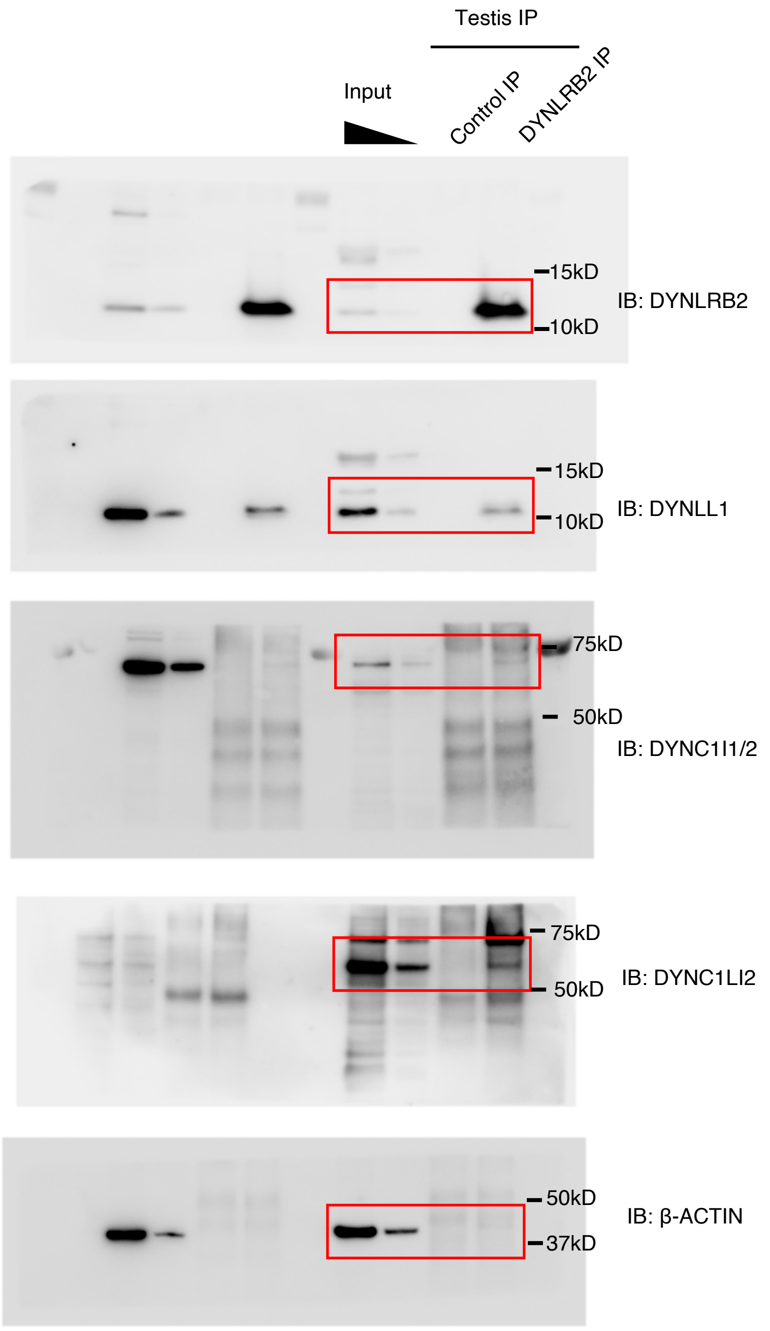

Source Data Supplementary Fig. 2a
Uncropped scans for Supplementary Fig. 2a

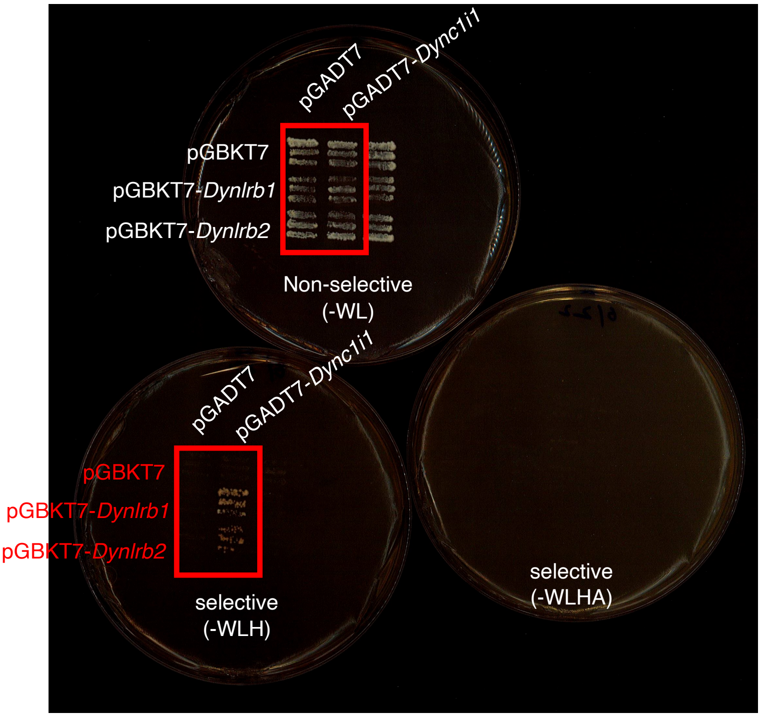

Source Data Supplementary Fig. 2c
Uncropped scans for Supplementary Fig. 2a
