# Supplementary material for: Distinct dynein complexes defined by DYNLRB1 and DYNLRB2 regulate mitotic and male meiotic spindle bipolarity
Source: Nat Commun. 2023 Mar 27;14:1715. doi: 10.1038/s41467-023-37370-7 (PMC10042829; doi:10.1038/s41467-023-37370-7)
Supplement: Supplementary file 3 — Description of Additional Supplementary Files [file 41467_2023_37370_MOESM3_ESM.pdf]

Description of Additional Supplementary Files

File Name: Supplementary Data 1
Description: The list of primers and siRNA sequences used in this study.

File Name: Supplementary Movie 1
Description: Single-molecule imaging of GFP-dynein immunoprecipitated with FLAG-hDYNLRB1.

File Name: Supplementary Movie 2
Description: Single-molecule imaging of GFP-dynein immunoprecipitated with FLAG-hDYNLRB2.
