# Supplementary material for: Distinct dynein complexes defined by DYNLRB1 and DYNLRB2 regulate mitotic and male meiotic spindle bipolarity
Source: Nat Commun. 2023 Mar 27;14:1715. doi: 10.1038/s41467-023-37370-7 (PMC10042829; doi:10.1038/s41467-023-37370-7)
Supplement: Supplementary file 7 — Reporting Summary [file 41467_2023_37370_MOESM7_ESM.pdf]

nature portfolio

Corresponding author(s): Hiroki Shibuya

Last updated by author(s): Mar 2, 2023

# Reporting Summary

Nature Portfolio wishes to improve the reproducibility of the work that we publish. This form provides structure for consistency and transparency in reporting. For further information on Nature Portfolio policies, see our Editorial Policies and the Editorial Policy Checklist.

## Statistics

For all statistical analyses, confirm that the following items are present in the figure legend, table legend, main text, or Methods section.

| n/a | Confirmed | |
|---|---|---|
| ☐ | ☒ | The exact sample size (*n*) for each experimental group/condition, given as a discrete number and unit of measurement |
| ☐ | ☒ | A statement on whether measurements were taken from distinct samples or whether the same sample was measured repeatedly |
| ☐ | ☒ | The statistical test(s) used AND whether they are one- or two-sided<br>*Only common tests should be described solely by name; describe more complex techniques in the Methods section.* |
| ☒ | ☐ | A description of all covariates tested |
| ☒ | ☐ | A description of any assumptions or corrections, such as tests of normality and adjustment for multiple comparisons |
| ☐ | ☒ | A full description of the statistical parameters including central tendency (e.g. means) or other basic estimates (e.g. regression coefficient) AND variation (e.g. standard deviation) or associated estimates of uncertainty (e.g. confidence intervals) |
| ☐ | ☒ | For null hypothesis testing, the test statistic (e.g. $F$, $t$, $r$) with confidence intervals, effect sizes, degrees of freedom and $P$ value noted<br>*Give P values as exact values whenever suitable.* |
| ☒ | ☐ | For Bayesian analysis, information on the choice of priors and Markov chain Monte Carlo settings |
| ☒ | ☐ | For hierarchical and complex designs, identification of the appropriate level for tests and full reporting of outcomes |
| ☒ | ☐ | Estimates of effect sizes (e.g. Cohen's *d*, Pearson's *r*), indicating how they were calculated |

*Our web collection on statistics for biologists contains articles on many of the points above.*

## Software and code

Policy information about availability of computer code

| | |
|---|---|
| Data collection | Images were obtained on a microscope (Olympus IL-X71 Delta Vision; Applied Precision) equipped with 100× NA 1.40 and 60× NA 1.42 objectives, a camera (CoolSNAP HQ; Photometrics), and softWoRx 5.5.5 acquisition software (Delta Vision). |
| Data analysis | Images were analyzed by softWoRx 5.5.5 acquisition software (Delta Vision). Pictures in Fig. 3e were processed with the deconvolution algorithm in softWoRx 5.5.5. All acquired images were processed with Photoshop (Adobe).<br><br>Sequence reads from PD5-25 WT mouse testis were aligned to the reference mouse genome data (mm10) using the 10x Genomics Cell Ranger count pipeline version 6.0.2 with the default settings, and the multiplexed samples were aggregated using the Cell Ranger aggr pipeline with default settings. Cell populations were clustered into seven groups by the k-means clustering method and plotted by uniform manifold approximation and projection (UMAP) using 10x Genomics Loupe Browser software version 6.2. |

For manuscripts utilizing custom algorithms or software that are central to the research but not yet described in published literature, software must be made available to editors and reviewers. We strongly encourage code deposition in a community repository (e.g. GitHub). See the Nature Portfolio guidelines for submitting code & software for further information.

## Data

Policy information about availability of data

All manuscripts must include a data availability statement. This statement should provide the following information, where applicable:

- Accession codes, unique identifiers, or web links for publicly available datasets
- A description of any restrictions on data availability
- For clinical datasets or third party data, please ensure that the statement adheres to our policy

The authors declare that the data supporting the findings of this study are available within the paper and its Supplementary Information. Single-cell RNA sequencing data of young mouse testes were obtained from previously published reports (E-code E-MTAB-6946). Source data are provided with this paper. All other data supporting the findings of this study are available from the corresponding author upon reasonable request.

## Human research participants

Policy information about studies involving human research participants and Sex and Gender in Research.

| | |
|---|---|
| Reporting on sex and gender | There is no human research participant in this study. |
| Population characteristics | There is no human research participant in this study. |
| Recruitment | There is no human research participant in this study. |
| Ethics oversight | There is no human research participant in this study. |

Note that full information on the approval of the study protocol must also be provided in the manuscript.

# Field-specific reporting

Please select the one below that is the best fit for your research. If you are not sure, read the appropriate sections before making your selection.

☒ Life sciences ☐ Behavioural & social sciences ☐ Ecological, evolutionary & environmental sciences

For a reference copy of the document with all sections, see nature.com/documents/nr-reporting-summary-flat.pdf

# Life sciences study design

All studies must disclose on these points even when the disclosure is negative.

| | |
|---|---|
| Sample size | For the analysis of testis and body weight ratios, three mice were used for each genotype.<br>For the fertility test, three pairs of male and female were analyzed for each genotype.<br>For cytological analysis using spermatocytes, a minimum of 32 nuclei were used to allow for statistical testing.<br>For the analysis of seminiferous tubules, a minimum of 749 seminiferous tubules were used to allow for statistical testing. |
| Data exclusions | No data was excluded. |
| Replication | Each conclusion in the manuscript was based on results that were reproduced in at least two independent experiments and in at least two independent mice of each genotype. |
| Randomization | Mice were categorized based on their genotypes. The genotypes were determined by PCR. Cells were categorized based on differential siRNA treatment (control siRNA vs Dynlrb1 siRNA). |
| Blinding | The investigators were not blinded to allocation during the experiments or to outcome assessment.<br>This is because the phenotypes were quite obvious that observer can be sure without blind test.<br>Further, the observer unbiasedly and carefully performed the quantification with enough sample number to make sure the conclusion. |

# Reporting for specific materials, systems and methods

We require information from authors about some types of materials, experimental systems and methods used in many studies. Here, indicate whether each material, system or method listed is relevant to your study. If you are not sure if a list item applies to your research, read the appropriate section before selecting a response.

## Materials & experimental systems

| n/a | Involved in the study |
| --- | --- |
| ☐ | ☒ Antibodies |
| ☐ | ☒ Eukaryotic cell lines |
| ☒ | ☐ Palaeontology and archaeology |
| ☐ | ☒ Animals and other organisms |
| ☒ | ☐ Clinical data |
| ☒ | ☐ Dual use research of concern |

## Methods

| n/a | Involved in the study |
| --- | --- |
| ☒ | ☐ ChIP-seq |
| ☒ | ☐ Flow cytometry |
| ☒ | ☐ MRI-based neuroimaging |

# Antibodies

### Antibodies used

The following primary antibodies were used: rabbit antibodies against DYNLRB2 (1:100 for IF; 1:1000 for WB; this study), DYNC1H1 (1:100 for IF; 1:1000 for WB; Proteintech; 12345-1-AP, 00080529), DYNC1I2 (1:100 for IF; BETHYL; A304-529A-T, 1), STIL (1:500 for IF; BETHYL; A302-442A, 1), DYNLL1 clone EP1660Y (1:100 for IF; 1:1000 for WB; Abcam; ab51603, GR3251355-4), KIF11 (Eg5) (1:100 for IF; Sigma; HPA010568, A118067), α-tubulin (1:1000 for IF; Abcam; ab18251, GR3406015-1), SYCP1 (1:1000 for IF; Abcam; ab15090, GR3184119-1), NuMA (1:100 for IF; Abcam; ab36999, GR296250-1), NuMA clone EP3976 (1:100 for IF; 1:1000 for WB; Abcam; ab109262, GR154119-10), γ-tubulin (1:500 for IF; Abcam; ab11317, GR3415347-1), pericentrin (PCNT) (1:300 for IF; Abcam; ab4448, GR3354375-2), and NDC80 (1:100 for IF; this study); mouse antibodies against MLH1 clone G168-15 (1:100 for IF; BD Biosciences; 51-1327GR, 4136717), β-actin clone AC-74 (1:1000 for IF; Sigma; A2228-200UL, 067M4856V), α-tubulin clone DM1A (1:1000 for IF; Abcam; ab7291, GR3398636-5), γ-tubulin clone TU-30 (1:500 for IF; Abcam; ab27074, GR3246908-22), PLK1 clone 35-206 (1:500 for IF; Abcam; ab17056, GR3260806-7), CENP-E (1:100 for IF; this study), DYCN1I1/2, clone 74.1(1:1000 for IF; Millipore; MAB1618, 3601722), SAS-6 clone 91.390.21 (1:250 for IF; Santa Cruz; Sc-81431, C3021), and DYNC1LI2 (1:100 for IF; 1:300 for WB; this study); goat antibodies against DCTN1 (P150) (1:100 for IF; Abcam; ab11806, GR3359155-3); sheep antibodies against BubR1 (1:100 for IF; Abcam; ab28193, GR3205690-18); rat antibodies against centrin 2 (CETN2) clone W16110A (1:300 for IF; BioLegend; 698602, B333787); and chicken antibodies against SYCP3 (1:5000 for IF; Hiroki Shibuya lab).

The following secondary antibodies were used:
Donkey Anti-Rabbit Alexa 488 (1:1000; Invitrogen; A21206, 2376850)
Donkey Anti-Rabbit Alexa 594 (1:1000; Invitrogen; A21207,2313074)
Donkey Anti-Mouse Alexa 594 (1:1000; Invitrogen; A21203, 2352146)
Donkey Anti-Mouse Alexa 488 (1:1000; Invitrogen; A21202, 2309139)
Goat Anti-Chicken Alexa 647 (1:1000; Invitrogen; A21449,1806124)
Donkey Anti-Rat Alexa 594 (1:1000; Invitrogen; A21209, 1807726)
Donkey Anti-Rat Alexa 488 (1:1000; Invitrogen; A21208, 1810450)
Donkey Anti-Goat Alexa 488 (1:1000; Invitrogen; A32814, UE286661)
Donkey Anti-Sheep Alexa 594 (1:1000; Invitrogen; A11016, 1017334)
Mouse TrueBlot® ULTRA: Anti-Mouse Ig HRP (1:1000; ROCKLAND; 18-8817-33, 39899)
Rabbit TrueBlot® ULTRA: Anti-Rabbit Ig HRP (1:1000; ROCKLAND; 18-8816-33, 43708)

### Validation

Antibodies generated in Shibuya Lab were validated by western blotting (WB) as well as immunofluorescence analysis (IF) using mouse testis samples.

The following commercial antibodies have been validated in the corresponding studies:

DYNC1H1 (Proteintech, 12345-1-AP): Mouse, WB and IF (PMID: 36218033)
DYNC1I2 (BETHYL, A304-529A-T): Human and mouse, IF and IP (manufacturer's website: https://www.thermofisher.com/antibody/product/DYNC1I2-Antibody-Polyclonal/A304-529A-T)
DYCN1I1/2 (Millipore, MAB1618): Human, WB (PMID: 25205765)
DYNLL1 (Abcam, ab51603): Human, IF, IP and WB (PMID: 22965910)
SYCP1 (Abcam, ab15090): Mouse,IF (PMID: 34075040)
MLH1 (BD Biosciences, 51-1327GR): Mouse, IF, (PMID: 32345962)
β-ACTIN (Sigma, A2228-200UL): Mouse, WB, (PMID: 24818823)
α-tubulin (Abcam, ab7291): Mouse, IF (PMID: 31397872)
α-tubulin (Abcam, ab18251): Mouse, IF (PMID: 24572510)
Centrin2 (BioLegend, 698602): Human, IF and WB (PMID: 26354417)
NUMA (Abcam, ab36999): Human, IF and WB (PMID: 24753406)
NUMA (Abcam, ab109262): Human, IF (PMID: 31782546)
DCTN1 (P150) (Abcam, ab11806): Human and mouse, IF and WB (PMID: 17932487)
γ-tubulin (Abcam, ab27074): Mouse, IF (PMID: 29136647)
γ-tubulin (Abcam, ab11317): Mouse, IF (PMID: 28935709)
Pericentrin (Abcam, ab4448): Human and mouse, IF (PMID: 25422492)
PLK1 (Abcam, ab17056): Mouse, IF and IP (PMID: 25533956)
STIL (BETHYL, A302-442A): Human, IF (PMID: 31820547)
SAS6 (A Santa Cruz, Sc-81431): Human, IF (PMID: 30517871)
BubR1 (Abcam, ab28193): Mouse, IF (PMID: 31111756)
KIF11 (Sigma, HPA010568): Human, IF (PMID: 25422374)

# Eukaryotic cell lines

Policy information about cell lines and Sex and Gender in Research

| | |
|---|---|
| Cell line source(s) | Mouse cell line: B16-F1 (Sigma, Cat#92101203-1VL)<br>Human cell line: HEK293 (Sigma, Cat#CB_85120602)<br>Human HeLa cell line expressing GFP-dynein HC (Morgan E DeSantis lab)<br>Mouse primary fibroblast isolated from WT and Dynlrb2 KO male mice |
| Authentication | These cell lines are authenticated in the company (Sigma) and published papers. The authentication procedures are shown in company's webcites. |
| Mycoplasma contamination | To avoid contamination during the experiments, we added 2.5 μg/ml Plasmocin (InvivoGen) to the medium. |
| Commonly misidentified lines (See ICLAC register) | There is no misidentified lines in this study. |

# Animals and other research organisms

Policy information about studies involving animals; ARRIVE guidelines recommended for reporting animal research, and Sex and Gender in Research

| | |
|---|---|
| Laboratory animals | We used WT and genetically modified mice (Dynlrb2 KO). Dynlrb2 KO mice were generated in this study. All WT and knockout mice were congenic with the C57BL/6J background. We used adult (2 months old) male mice for most of the experiments, otherwise indicated in the figure legends. |
| Wild animals | No wild animal was used. |
| Reporting on sex | We studied the progression of male germ cell development. Thus, most of the data were obtained by using male mice. We used female mice only for the breeding and fertility assay. |
| Field-collected samples | Our study did not involve samples collected from the field |
| Ethics oversight | All animal experiments were approved by the Regional Ethics Committee of Gothenburg, governed by the Swedish Board of Agriculture (#1316/18). |

Note that full information on the approval of the study protocol must also be provided in the manuscript.
